# Temperature-driven shifts in foraging behaviour during larval development in a dragonfly

**DOI:** 10.1038/s41598-026-37523-w

**Published:** 2026-02-04

**Authors:** Jolan Hogreve, Frank Johansson, Frank Suhling

**Affiliations:** 1 Landscape Ecology and Environmental Systems Analysis, Institute of Geoecology, Technische Universität Braunschweig, Braunschweig, Germany; 2 Department of Ecology and Genetics, Animal Ecology, Evolutionary Biology Centre, Uppsala University, Uppsala, Sweden

**Keywords:** Foraging behaviour, Temperature, Ontogeny, Odonata larvae, Learning, Ecology, Ecology, Evolution, Zoology

## Abstract

Predatory performance of dragonfly larvae is influenced by a multifaceted interplay of external factors such as temperature, prey density and interspecific competition, and life history traits like age and size. We investigated the relative impact of these factors and traits on the prey-capture behaviour of *Sympetrum striolatum* larvae i.e., the number of strikes, captures and capture success. The larvae were observed three times over a five-week period under a combination of three temperature levels, two prey densities, and with or without a conspecific competitor. To access the ontogenetic effects on foraging behaviour the larvae were reared from hatching and their size measured before each trial. Higher temperature, particularly for young and small larvae, and prey density significantly increased prey-capture behaviour. The life history traits strongly affected strikes, captures, and capture success and these effects were stronger than the external factor prey density or competition. These results underscore the crucial role of ontogeny on foraging performance. Future studies and predictive models of foraging behaviour should incorporate life history to better understand foraging dynamics. Our study highlights the importance of integrating developmental biology into understanding behaviour under environmental change, rather than focusing solely on external variables.

## Introduction

Ongoing anthropogenic climate change, particularly warming, affect species behaviours^1^, which among other drivers can affect intraspecific and interspecific interactions^2,3^, ultimately leading to shifts in population and community dynamics^4,5^. Such effects may be especially important for ectotherms. These organisms are particularly susceptible to climate warming as their physiological processes rely on external temperatures^6^. Higher temperatures influence metabolism by increasing energy demands^7^. As a result, behaviour is highly sensitive to changes in temperature^8,9^. One primary behavioural trait that has been shown to be important for the dynamics of population and communities is foraging behaviour^10^. Foraging behaviour includes food intake which is important for growth and development which in turn affects important fitness components^11^. For example, bumblebee workers reared at 33 °C had higher visiting rates and shorter visiting times at flowers than those reared at 27 °C^12^. Also, the duration of ants’ foraging trips decreased with increasing temperature, which led to an increased foraging speed as the ants approached their critical temperature. However, the distance travelled did not change^13^. However, the increased foraging behaviour as a result of higher temperatures may be offset by negative effects. It has been shown that rising temperatures can lead to challenges such as altered competition dynamics or higher mortality rates^14,15^. These outcomes might reflect complex trade-offs and interactions that remain insufficiently understood.

Foraging behaviour also changes throughout ontogeny^16,17^. Animals adapt their behaviour to complex environmental conditions, balancing the costs and benefits to their fitness^18^. Consequently, changes in behaviour during development can directly impact an organism’s ability to acquire resources and evade predators, ultimately affecting its fitness and population dynamics. Since behaviour is affected by temperature^19^, studying the interaction between temperature, development and behaviour could provide important insights into how potential climate change can affect behaviours such as foraging.

Dragonflies are valuable indicators of environmental conditions^20^ connecting freshwater and terrestrial communities^21^. The larvae act as intermediate predators^22^ and foraging activity is one of the key drivers for their population structure as they interact in strong inter- and intraguild predation^23^. While the metabolic and physiological temperature response are well studied and known to influence life histories^24^, growth^25^, functional response^26,27^ and trophic interactions^28–30^, direct observations of temperature dependant behaviour may offer deeper mechanistic insights into these processes.

The purpose of this study was to examine the effects of three different temperatures on foraging behaviour of dragonfly larvae considering life history traits. Food availability was included as an additional factor since higher temperatures lead to increased energy requirements. Furthermore, behaviour is affected by intraspecific competition in general^31,32^ and specifically for dragonfly larvae^33,34^. Therefore, we also investigated how the presence of a conspecific competitor affects foraging behaviour. By examining behaviour with and without a potential competitor across temperature conditions, we aimed to better understand how competition dynamics shift in response to temperature changes. We studied three different foraging behaviours: strike at prey, capture of prey and capture success. We predicted that: (1) higher temperatures would lead to an increase in strike frequency and capture rate due to accelerated metabolic processes; (2) the presence of a competitor would reduce strike frequency, capture rate, and capture success, as interference competition is expected to divert attention and disrupt prey-capture behaviour; and (3) an increase in capture success over developmental time, which would reflect the maturation of neural networks, sensory-motor integration and learning as larvae develop and grow.

## Results

### Foraging behaviour

The experiments showed that the presence of a second larva as competitor only had a limited effect on the foraging behaviours. The competitor had a minor or no significant effect on the number of strikes and captures depending in the model (Table 1) or capture success. Furthermore, there was no measurable effect of direct interaction between the larvae in any of the behavioural experiments; no larvae were attacked, killed or eaten. Therefore, competition as a factor was excluded from the figures.

**Table 1:** GLM results for the effects of age or size, food density, temperature and competition on number of strikes and captures per three-minute interval, including ΔAIC values comparing the null and final model.

		Fixed Effects	Estimate	SE	z-value	p
Age based model	Strikes	*Intercept*	2.965	0.087	33.99	< 0.001
		Age	0.703	0.061	11.53	< 0.001
		Age²	−0.169	0.045	−3.75	< 0.001
		Temperature 22 °C	0.454	0.082	5.51	< 0.001
		Temperature 28 °C	0.333	0.083	4.03	< 0.001
		Food Density Low	−0.416	0.067	−6.24	< 0.001
		Competition Individual	0.125	0.067	1.88	0.06
		Age * Temperature 22 °C	−0.451	0.084	−5.39	< 0.001
		Age * Temperature 28 °C	−0.670	0.084	−8.00	< 0.001
		Δ AIC	139.15			
	Captures	*Intercept*	2.572	0.088	29.16	< 0.001
		Age	1.069	0.067	15.87	< 0.001
		Age²	−0.252	0.045	−5.58	< 0.001
		Temperature 22 °C	0.679	0.085	7.95	< 0.001
		Temperature 28 °C	0.619	0.085	7.26	< 0.001
		Food Density Low	−0.441	0.066	−6.65	< 0.001
		Competition Individual	0.129	0.066	1.94	0.05
		Age * Temperature 22 °C	−0.601	0.088	−6.83	< 0.001
		Age * Temperature 28 °C	−0.852	0.088	−9.70	< 0.001
		Δ AIC	255.66			
Size based model	Strikes	*Intercept*	1.760	0.147	11.94	< 0.001
		Size	1.043	0.120	8.68	< 0.001
		Size²	−0.118	0.028	−4.16	< 0.001
		Temperature 22 °C	0.687	0.192	3.58	< 0.001
		Temperature 28 °C	0.699	0.221	3.16	0.002
		Food Density Low	−0.404	0.071	−5.71	< 0.001
		Competition Individual	0.093	0.071	1.31	0.18
		Size * Temperature 22 °C	−0.317	0.107	−2.97	0.003
		Size * Temperature 28 °C	−0.389	0.116	−3.37	< 0.001
		Δ AIC	94.60			
	Captures	*Intercept*	0.751	0.157	4.78	< 0.001
		Size	1.611	0.124	12.98	< 0.001
		Size²	−0.184	0.029	−6.40	< 0.001
		Temperature 22 °C	0.818	0.202	4.06	< 0.001
		Temperature 28 °C	0.763	0.232	3.30	< 0.001
		Food Density Low	−0.427	0.072	−5.89	< 0.001
		Competition Individual	0.089	0.073	1.22	0.22
		Size * Temperature 22 °C	−0.399	0.110	−3.63	< 0.001
		Size * Temperature 28 °C	−0.446	0.119	−3.76	< 0.001
		Δ AIC	176.89			

**Fig. 1:**
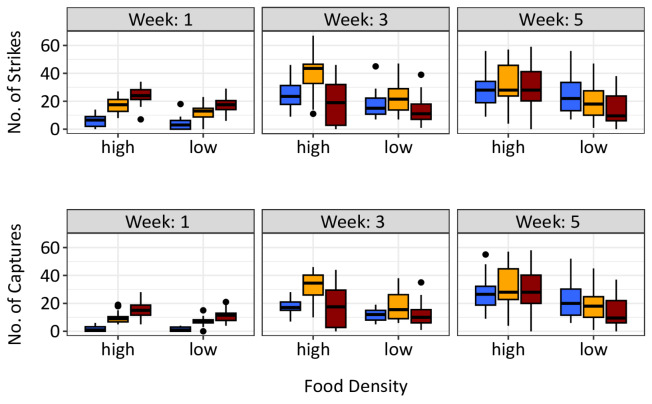
Number of strikes and captures for 16 °C (blue), 22 °C (orange) and 28 °C (red) at high and low food density.

**Fig. 2:**
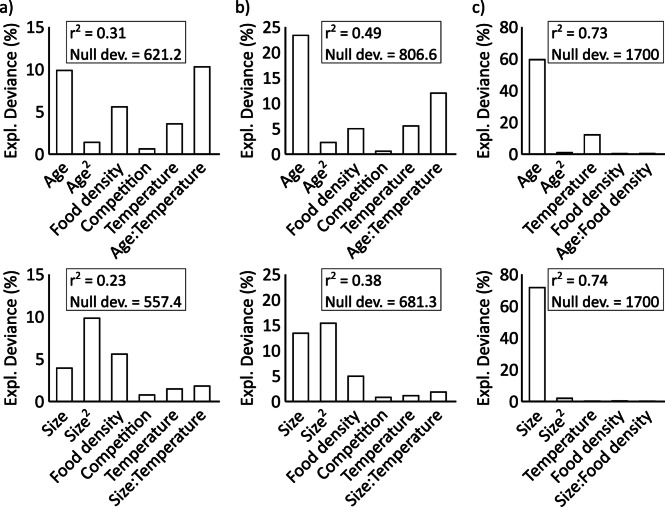
Relative contribution of the individual factors to the explained deviance in the generalized linear models (GLMs) for (**a**) strikes, (**b**) captures and (**c**) success rate (%). Top panels represent age-based models, bottom panels represent size‐based models indicating the percentage contribution (%) of each factor/trait to the explained deviance of each model, including the deviance of the corresponding null model (df_null_ = 359) and the models pseudo-r².

The results of strikes and captures were affected significantly by age, temperature and food density (Figs. 1 and 2; Table 1). As age progressed, the number of strikes and captures exhibited a notable increase, indicating a significant age effect. A significant quadratic age effect was found suggesting that the increase with age was non-linear. However, at week 5 number of strikes and captures did not differ between temperatures at high prey density but declined with increasing temperature for low prey density (Fig. 1). Temperature had a significant positive effect on the number of strikes and captures. Higher temperatures were associated with an increase in the number of strikes as well as an even more pronounced increase in captures. The significance of the interaction term between age and temperature is particularly noteworthy. This implies that the effect of age was strongly influenced by temperature in the model. As the temperature increased, the age effect became less pronounced, as observed in the differences between weeks depicted in Fig. 1. Compared to a high food density, a reduced food density had a negative effect on number of strikes and captures, as indicated by a significant food effect (Fig. 1; Table 1). The number of strikes and catches depending on size is shown in Fig. 4 and the model that utilised size as a factor instead of age produced qualitatively similar result for strikes and captures (Fig. 2; Table 1). Comparing the two models, the models for age had a higher explained deviance and r² (Fig. 2).

**Fig. 3:**
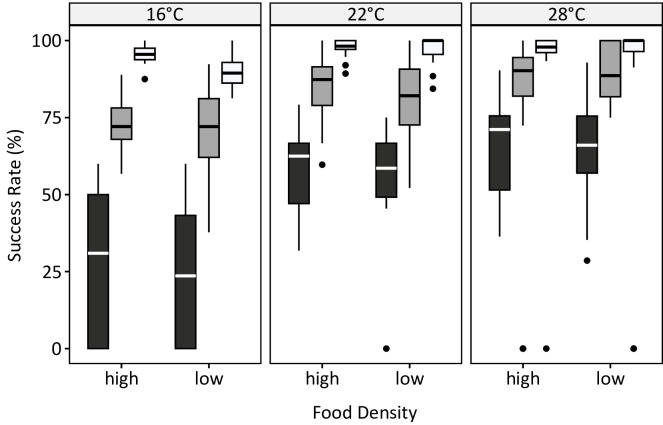
Capture success rate (%) of larvae at 16 °C, 22 °C and 28 °C for high and low food density over time (black = week 1, dark grey = week 3, light grey = week 5).

**Fig. 4:**
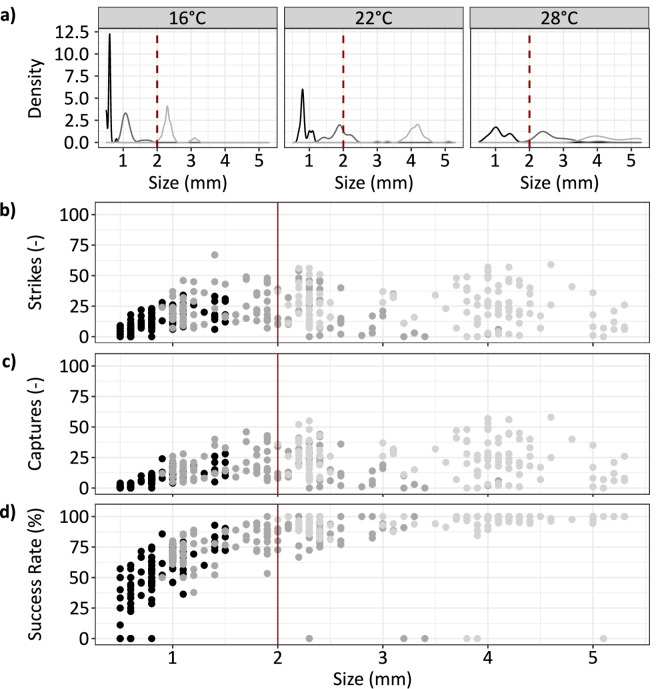
(**a**) Distribution of larval head size (mm) over time, coloured according to time (black = week 1, dark grey = week 3, light grey = week 5 and red lines = 2 mm head width) and (**b**) strikes, (**c**) captures and (**d**) success rate (%) over size (mm).

The success rate was significantly affected by temperature, age and food density (Table 2). Similar to strikes and captures, a significant quadratic effect of age was detected, indicating that the age-related increase was non-linear. Temperature had a significant effect, with larvae showing a higher success rate at higher temperatures (Fig. 3). Also, success rates increased significantly with ongoing age, i.e., over time at all temperatures. The success rates exhibited a continuous upward trend, surpassing 90% by week 5 and ultimately reaching mostly 100% as the larvae aged (Fig. 3). A significant effect of food density was also observed (Fig. 3; Table 2), and the significant interaction term between age and food density suggested that the age effect decreased when the food density was low. When size was used as the determining factor in the model instead of age, the qualitative results of the analysis remained the same, except for temperature and food density, which were not significant in this case (Fig. 2; Table 2). The model with size had a higher explained deviance and a minimal higher r² compared to the model using age as factor (Fig. 2). Once the larvae surpassed a size of approximately 2 mm, there was no strong further increase in success rates, which reached a maximum of 75–100% (Fig. 4). Larval size depended on both age and temperature: larvae at 16 °C required five weeks to reach this size, whereas those at 28 °C achieved it in the third week of the study (Fig. 4).

**Table 2:** GLM results for the effects of age or size, food density, temperature and competition on the success rate (captures/strikes) per three-minute interval, including ΔAIC values comparing the null and final model.

	**Fixed Effects**	**Estimate**	**SE**	**z-value**	**p**
Age based model success rate (%)	*Intercept*	0.888	0.072	12.33	< 0.001
	Age	1.390	0.059	23.67	< 0.001
	Age²	0.101	0.048	2.13	0.03
	Food Density Low	−0.230	0.071	−3.23	< 0.001
	Temperature 22 °C	0.845	0.082	10.29	< 0.001
	Temperature 28 °C	1.258	0.093	13.60	< 0.01
	Age * Food Density Low	−0.205	0.080	−2.60	< 0.01
	Δ AIC	1236.80			
Size based model success rate (%)	*Intercept*	−1.24	0.15	−8.30	< 0.001
	Size	2.02	0.15	13.92	< 0.001
	Size²	−0.19	0.03	−6.16	< 0.001
	Food Density Low	−0.01	0.14	−0.08	0.93
	Temperature 22 °C	−0.11	0.08	−1.44	0.15
	Temperature 28 °C	−0.15	0.08	−1.81	0.07
	Size * Food Density Low	−0.10	0.08	−1.24	0.21
	Δ AIC	1251.03			

### Survival

**Fig. 5:**
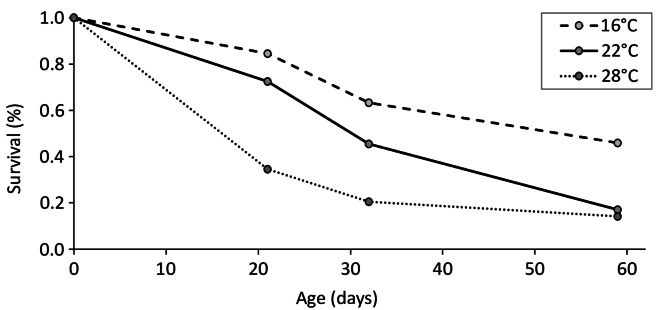
Survival (%) for the larvae at 16 °C, 22 °C and 28 °C during the entire rearing period.

Not all larvae survived the rearing process. Dead larvae were systematically removed from the cage, and some were found with feeding marks. Survival rates varied significantly with temperature (contingency table, chi^2^ = 83.815, df = 6, *p* < 0.001). Overall survival rates were higher at low temperatures than at high temperatures. Survival was 45.8% at 16 °C, 17.1% at 22 °C and 14.2% at 28 °C. While mortality at 16 °C and 22 °C showed a nearly linear trend over time it is notable that the mortality at 28 °C was initially very high, before dropping to a relatively constant low level (Fig. 5).

## Discussion

We hypothesized that an increase in temperature would lead to an increase in the intensity of the foraging behaviour of dragonfly larvae. Our findings supported this hypothesis, as the number of strikes, the number of captures, and the foraging success rate varied depending on temperature. Especially for the success rate, increased temperatures lead to increased hunting success. However, we acknowledge that the situation is more complex. We found that foraging behaviour changed during ontogeny, i.e., with larvae’s age and size (which correlate), and that these traits interacted with temperature.

### Temperature

Although various components of larval behaviour such as foraging and activity have been widely studied^35,36^, less is known about how temperature affects such behaviours. In the study most comparable to ours, temperature had no significant effect on any of the behaviours investigated (i.e., prey capture success, activity and boldness) in *Sympetrum fonscolombii* and *S. vulgatum*^37^. However, that study focused on only two temperatures, which were in the lower range compared to ours. Other previous studies on aquatic insects have also found a higher capture success at higher temperatures. For example, capture success of *Notonecta* on tadpoles increased with temperature^38^ or capture success of damselfly larvae of *Enallagma annexum* increased with temperature^39^.

The numbers of strikes and captures increased with rising temperature in the first week. This may imply that 28 °C was not yet beyond the species-specific optimum temperature, since T_opt_ for growth in younger larvae is 28.4 °C^25^. Our observed result may simply reflect the requirements of more food due to the higher metabolism at higher temperature. Interestingly, we found that the influence of temperature varied over time. After five weeks, the number of strikes and captures were lower at the higher temperatures suggesting the presence of an optimal temperature range for foraging behaviour. Several factors could explain this phenomenon. For instance, acclimation to temperatures may have occurred, leading to a decrease in the behavioural differences observed at the beginning of the experiments^40^. Alternatively, at elevated temperatures, larvae may experience chronic stress^41^ potentially leading to a decrease in the frequency of strikes and captures. This aligns with higher mortality at higher temperatures.

Foraging success was also higher at higher temperatures, but in contrast to strikes and captures it did not decline over time. In fact, it increased, reaching its peak at week 5. This suggests that temperature is not the only parameter affecting foraging success (see below). Other studies have found no significant effect of temperature on behaviour but have also identified intricate patterns between behaviour and larval ontogeny^37^.

There are several reasons that could contribute to the improvement in capture success over time observed in our experiment. One potential explanation is the refinement and coordination of neural networks and associated sensory feedback mechanisms as the larvae develop. Enhanced cognitive abilities would allow more efficient and effective foraging behaviours^42^. An additional explanation for the increase in capture success includes the involvement of a learning process^43,44^. The ability to learn and behave adaptively can directly influence fitness^45^ and probably exists in all animals^46^. Although research has primarily focused on invertebrates, particularly social insects^47,48^, there are also descriptions of adaptive behaviour and learning in non-social insects^49^. For example, damselfly (Zygoptera) larvae can recognise the presence of fish predators through chemical cues^50^. Furthermore, *Anax imperator* larvae display changes in prey selection when encountering familiar prey items^43^.

In our study, the success rate changed synchronously over time at all three temperatures, although the success rate did not reach 100% at 16 °C. This suggests that, despite varying larval development rates, a consistent pattern of hunting success emerges. This pattern can be interpreted as a case of behavioural adaptation. The larvae would have gained experience in prey capture strategies, aligning with the definition of learning as an adaptive change in individual behaviour resulting from prior experience^51^. Furthermore, changes in size may alter the success rate (see below).

### Survival

Survival was also temperature-dependent, with the lowest survival rate occurring at the highest temperature. This is consistent with other observations that high temperatures increase the mortality of odonate larvae^52,53^. This pattern is sometimes explained by ambient temperature reaching the upper thermal limit of survival. However, thermal limits of odonate larvae are all above 30 °C^25^. The highest temperature in our experiment was below this threshold, but close to the growth optimum (see above). We suggest that nutritional deficiency resulting from by a relatively one-sided diet could be the driving force behind the higher mortality at 28 °C. Since metabolism increases with rising temperature, the demand for food is also expected to increase^25,54^. This has been demonstrated in functional response studies conducted at different temperatures^26,55,56^. Mortality in the storage containers, particularly at 28 °C, was high but levelled off after around 30 days. This suggests that high temperatures may affect small *Sympetrum* larvae more severely, either physiologically or by cannibalism. Therefore, it is interesting to consider not only overall mortality, but also stage-related mortality.

### Food density

Predatory behaviour is condition-dependent and influenced by various factors, including hunger and exhaustion^57^, as well as larval size, weight and ambient temperature^56^. A sufficient food supply is crucial for larval development^58^, and larvae tend to remain in areas with high food supply^59^. Prey density, prey size, prey behaviour as well as the predator-prey size ratio can strongly affect the behaviour and capture success^60^. For example, Hirvonen & Ranta^61^ found that larger larvae of *Aeshna juncea* improved their intake of biomass as the size and density of their prey increased. The results of our study align with these findings. At lower food density, observed behaviours and success rates were lower. This reduction allocated for striking and capturing may be attributed to the increased time spent searching for prey. Conversely, high food density provided larvae with abundant and accessible prey items, enabling them to hunt food efficiently.

While high prey densities in the final week of the experiment demonstrated a saturation effect in strikes and captures, a decline was observed for low prey densities (i.e., for largest larvae). The largest larvae most likely becoming less motivated to hunt *Artemia*, especially at low densities suggesting it being not the optimal prey at this stage of development^25^. Nevertheless, we observed instances of larvae, independent of their age and size, that did not hunt at all during the trials. Such variation in behaviour among individuals may be indicative of divergent energy requirements or individual strategies.

### Competition

We found only a weak to no effect of competition on foraging behaviour. Dragonfly larvae can act as both predators and prey within the same habitat, depending on size differences which is referred to as size-mediated intraguild predation^23^. If these size differences are substantial, they can act as predators; otherwise, they compete directly for resources^23,54,62^. Consequently, we hypothesized that the presence of a competitor would lead to behavioural modifications, such as decreased foraging activity^22^. At a more mechanistic level, we anticipated that the presence of a conspecific would lead to a conflict of interest between the need to increase (foraging) activity in response to rising temperatures and the need of interaction avoidance-behaviour^63^. Since high feeding rates are generally linked with rapid development^59,64^, a reduction in feeding would pose a significant disadvantage. Therefore, we predicted that foraging effort and, thus, interaction strength would increase with a rise in temperature. However, our results did not support this expectation. One possible explanation is that we only had a single conspecific competitor. Stronger competition may typically emerge at higher larval densities, where resource scarcity is more pronounced^33,66^.

### Size

During our experiments, larval development was directly affected by ambient temperature, as a result of the temperature-growth response^25^. This resulted in a rapid deviation in size between the temperature treatments. At week 1, the larvae in all temperature treatments exhibited relatively similar sizes. However, by week 3 and 5, those reared at 22° and 28 °C were larger than those reared at 16 °C. Consequently, all larvae used in our experiments were the same age, but their size varied depending on the rearing temperature. Smaller larvae may exhibit a lack of handling ability or efficiency as indicated by the lower success rates in the beginning, or they are faster satisfied and consequently consume less *Artemia* than their larger counterparts. Therefore, the observed pattern of the hunting behaviour and success may be influenced by the age and size of the larvae, i.e., the stage of their development^65^. For instance, small larvae of *A. juncea* in field experiments exhibit reduced activity to avoid the encounters with larger conspecifics^66^, or generally modify their foraging behaviour^67^. Also, the larvae of *A. junius* were described to display age-specific behaviour^68^. In our experiment, we found also that larger sized larvae have a higher foraging success rate with less variation between individuals. This increased success may be directly related to size, encompassing factors such as temperature-dependent movement speed, the relationship between the labium and the size of the prey, and improved sensory perception, such as the development of the eyes. The eyes of dragonfly larvae continue to develop throughout their larval stages, becoming increasingly complex^69,70^. Consequently, visual prey detection generally improves as they mature^71^. Given this developmental trajectory, our results show that hunting success is closely associated not only with developmental stage and experience, but also with size.

## Conclusion

Our study indicates that increasing temperature affects the foraging behaviour of dragonfly larvae. Furthermore, age and size clearly influence behaviour and interact with the temperature effect. This suggests that larvae do not exhibit consistent behaviour throughout their development but rather show plastic behaviour, varying e.g., with age and size. These findings are crucial for gaining a deeper comprehension of the ecological dynamics and evolutionary biology of these organisms, particularly in the context of rising environmental temperatures.

## Methods

### Study species

The objective of this study was to assess foraging behaviour and life history traits of *Sympetrum striolatum* (Charpentier, 1840) larvae, a widespread dragonfly species in the western Palaearctic and east towards Japan. The species inhabits both permanent and temporary standing waters, and sometimes running waters^72^. In Central Europe, *S. striolatum* exhibits a marked population increase, which is assigned to climate change^73^.

### Pre-treatments

The larvae were reared from six egg clutches collected on October 29, 2021 from a population in Braunschweig, Germany. The eggs were obtained by dipping the female’s abdomen into water^74^. To delay embryogenesis^75^, we overwintered eggs by keeping them at 5 °C in darkness in a fridge until 22th April 2022. They were then transferred to Uppsala University, where hatching started on April 30, 2022. On May 02, 2022 the freshly hatched larvae from all clutches were mixed and transferred into 0.7 L plastic containers filled with 300 ml of non-chlorinated tap water. Sera©Mycopur (one drop per two litres) was added to the water to prevent fungal infection. Small grass clippings and a piece of plastic mosquito net were added to provide a clinging structure. All larvae were reared in a climate chamber with a controlled environment set to 16 °C and a 14:10 h light: dark photoperiod. EHEIM aquarium heaters were used to set two higher water temperatures of 22 °C, and 28 °C, generating the three experimental temperatures: 16, 22 and 28 °C. Temperature never varied more than 0.5 °C on a daily basis. For each temperature, 240 larvae were reared in groups of ten larvae per container. The larvae were fed daily with brine shrimp (*Artemia* sp. naupliae from laboratory cultures), which were provided *ad libitum*. They were reared for one week at their respective temperature until the start of the experiments. Total larval survival was recorded on days: 1, 22, 33 and 60 and expressed in % of total number of larvae.

### Experimental treatments

The larvae were starved for 24 h before each observation. Before each observation run the larval size was measured by photographing them from a dorsal view. We measured the maximum head width in mm by using ImageJ^76^. Each experimental setup was assigned a fixed container of larvae, ensuring the same group of individuals was used consistently across the repeated trials.

Behavioural observations were carried out in 90 mm diameter Petri dishes filled with non-chlorinated tap water. The effects of three factors on larval behaviours were investigated: temperature, food density and competition by a conspecific. The temperature treatments were here also 16, 22, and 28 °C. Food (*Artemia* sp. naupliae) was provided at low and high densities. For this purpose, an *Artemia* solution containing approximately 100 or 400 *Artemia* sp. naupliae in 50 ml with the respective water temperatures were prepared, representing low and high food densities. The solution was then added to the Petri dish. One single larva (no competition treatment) or two larvae of a similar size (competition treatment) were introduced. The potential competitor was marked on the leg and came from a different container to exclude the possibility that the larvae had become accustomed to each other beforehand. Only the behaviour of the determined and not marked larva was recorded. No *Artemia* mortality was observed, except for individuals consumed by the larvae.

All observation of behaviours were made under a dissecting microscope for three minutes. The number of strikes and the number of captures was counted. The experiment was conducted a total of 360 times, 40 times per temperature treatment per week. From these two behaviours we then calculated a third behaviour: the success rate (%) by dividing the number of total strikes by the number of successful strikes.

Behavioural observations were carried out three times at two-week intervals: one week after hatching (week 1), and then at weeks 3 and 5. As it was not feasible to observe the behaviour of all larvae for all variable combinations in a single day, observations were carried out over five consecutive days during each observation period (week 1, 3, and 5) This provided us with an additional information of the age of the larvae at the time of observation. In the statistical analyses, age was defined as the number of days since hatching.

### Analyses

The statistical open-source software R^77^ extended with the ‘MASS’^78^ and ‘lme4’^79^ packages were applied for the statistical analyses. Generalized linear models (GLMs) were used to analyse the effects of age, size, temperature, food density and competition on the number of strikes, captures and success rate. In our experiment a single container was always assigned to a specific setup. Following this, the container-ID should be included as a random factor in the statistical models. After testing it, it turned out to be near zero or zero and therefore it was dropped as being negligible^80^.

A negative binomial distribution was used for modelling to handle the dispersion of captures and strikes. A binomial model was used to estimate the success rate. All models included quadratic terms for the standardised age or size considering the non-linear relationships. We included larval age or size as dependent variables in the models but performed separate models for age and size. The reason for using separate models was to handle collinearity as size and age were strongly correlated (Pearson: r^2^ = 0.84, *p* < 0.001). Every possible combination of the predictors (including their interactions) was tested and model performance was compared using the Akaike information criterion (AIC). The model with the combination of variables showing the lowest AIC value, indicating the best trade-off between goodness-of-fit and model complexity was selected. The ΔAIC between the intercept-only model (null model) and the final model was calculated as a comparative value and McFaddens pseudo-R² was calculated as indicator of model fit. In order to examine the impact of the predictors in the final model for each behaviour, an ANOVA Sum of Squares Type III was performed and bar-plots used for illustrating each factor or traits relative contribution to the observed deviance. Furthermore, we used a chi²-test to compare the mortality of the reared larvae at the three different temperatures.

## Data Availability

Raw data will be available at LeoPARD [https://leopard.tu-braunschweig.de/].
